# Crystal structure of the high-energy-density material guanylurea dipicryl­amide

**DOI:** 10.1107/S1600536814017164

**Published:** 2014-07-31

**Authors:** Raik Deblitz, Cristian G. Hrib, Liane Hilfert, Frank T. Edelmann

**Affiliations:** a Chemisches Institut, Otto-von-Guericke-Universitaet Magdeburg, Universitaetsplatz 2, D-39106 Magdeburg, Germany

**Keywords:** crystal structure, high-energy-density material, guanylurea dipicryl­amide, hydrogen bonding, dipicryl­amides, energetic compounds, guanylurea salts

## Abstract

The title compound, 1-carbamoylguanidinium bis­(2,4,6-tri­nitro­phen­yl)amide [H_2_NC(=O)NHC(NH_2_)_2_]^+^[N{C_6_H_2_(NO_2_)_3_-2,4,6}_2_]^−^ (= guanylurea dipicryl­amide), was prepared as dark-red block-like crystals in 70% yield by salt-metathesis reaction between guanylurea sulfate and sodium dipicryl­amide. In the solid state, the new compound builds up an array of mutually linked guanylurea cations and dipicryl­amide anions. The crystal packing is dominated by an extensive network of N—H⋯O hydrogen bonds, resulting in a high density of 1.795 Mg m^−3^, which makes the title compound a potential secondary explosive.

## Abstract 2

1-Carbamoylguanidinium bis­(2,4,6-tri­nitro­phen­yl)amide (= guanylurea dipicryl­amide) builds up an array of mutually linked guanylurea cations and dipicryl­amide anions. The crystal packing is dominated by an extensive network of N—H⋯O hydrogen bonds, resulting in a high density of 1.795 Mg m^−3^ which makes the title compound a potential secondary explosive.

## Chemical context   

High-energy-density materials (HEDMs) form an important class of explosive compounds. Several significant advantages such as high heats of combustion, high propulsive power, high specific impulse, as well as smokeless combustion make them highly useful as propellants, explosives, and pyrotechnics (Oestmark *et al.*, 2007 ▶; Rice *et al.*, 2007 ▶; Badgujar *et al.*, 2008 ▶; Göbel & Klapötke, 2009 ▶; Nair *et al.*, 2010 ▶; Klapötke, 2011 ▶). An important class of such high-energy-density materials are polynitro aromatics such as tri­nitro­toluene (TNT), picric acid, tri­nitro­resorcinol (= styphnic acid), and 2,2′,4,4′,6,6′-hexa­nitro­diphenyl­amine (= dipcryl­amine). Dipicryl­amine com­bines several very inter­esting structural features: It contains six nitro groups, which are flexible and can inter­act and adjust in the crystal lattice. Moreover, dipicryl­amine has a secondary amine group which can be deprotonated with alkali and alkaline earth-metal hydroxides to form water-soluble dipicryl­amide salts. In the resulting dipicryl­amide anion (= DPA^−^), partial delocalization of the negative charge mediated by the aromatic rings is possible, which may facilitate coord­ination of the oxygen atoms of the nitro groups with suitable metal ions (Eringathodi *et al.*, 2005 ▶; Agnihotri *et al.*, 2006 ▶). Moreover, the DPA^−^ anion has various sites which are capable of forming different types of hydrogen bonds in the solid state.
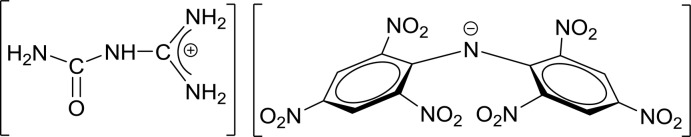


The ammonium salt of dipicryl­amine, also known as Aurantia or Imperial Yellow, was discovered in 1874 by Gnehm and used as a yellow colorant for leather, wool, and silk until the early 20^th^ century (Gnehm, 1874 ▶, 1876 ▶). However, these practical uses have been terminated due to the highly toxic and explosive nature of dipicryl­amine (Kjelland, 1971 ▶). Dipicryl­amine can also be used for the extraction of K^+^ ions from sea bittern, which contains a mixture of K^+^, Na^+^, and Mg^2+^ salts (Winkel & Maas, 1936 ▶). A related study carried out with a mixture of K^+^, Rb^+^, and Cs^+^ ions revealed that the Cs^+^ ion shows maximum selectivity towards DPA^−^ (Bray *et al.*, 1962 ▶). In fact, it has been reported that DPA^−^ can be used for the recovery of Cs^+^ from radioactive wastes (Kyrš *et al.*, 1960 ▶). Only in recent years has the structural chemistry of alkali metal and alkaline earth metal as well as ammonium and azolium dipicryl­amides been investigated in detail. All these compounds were found to display inter­esting hydrogen-bonded supra­molecular structures in the solid state (Eringathodi *et al.*, 2005 ▶; Agnihotri *et al.*, 2006 ▶; Huang *et al.*, 2011 ▶).

## Spectroscopic features   

In the course of our ongoing studies on the crystal structures of energetic compounds (Deblitz *et al.* 2012*a* ▶,*b* ▶; Stock *et al.*, 2014 ▶), we investigated the preparation and structural characterization of the title compound, guanylurea dipicryl­amide, [H_2_NC(=O)NHC(NH_2_)_2_]_2_[N{C_6_H_2_(NO_2_)_3_-2,4,6}_2_]. The guan­yl­urea cation has frequently been reported to be a useful component in energetic nitro­gen-rich salts, *e.g.* guanylurea dinitramide (Langlet, 1998 ▶) or guanylurea tetra­zolate salts (Wang *et al.*, 2009 ▶). An aqueous solution of sodium dipicryl­amide was prepared *in situ* by deprotonating dipicryl­amine with NaOH. Treatment of this dark-red solution with solid 1-carbamoylguanidinium sulfate, [H_2_NC(=O)NHC(NH_2_)_2_]_2_SO_4_ (also known as guanylurea sulfate) (Lotsch & Schnick, 2005 ▶), afforded dark-red block-like crystals of the title compound after undisturbed standing of the reaction mixture for 10 d. The product was characterized by spectroscopic methods and elemental analysis. The ^1^H NMR spectrum displayed a sharp singlet at δ = 8.78 p.p.m. for the aromatic protons of the DPA^−^ anion, which is in excellent agreement with the literature values (Eringathodi *et al.*, 2005 ▶; Agnihotri *et al.*, 2006 ▶; Huang *et al.*, 2011 ▶). However, the NH and NH_2_ protons only gave rise to two very broad resonances spread over a range of *ca* 4 p.p.m. [δ(C(O)NH_2_] = *ca* 6.3–7.1 p.p.m., δ[NHC(NH_2_)_2_] = *ca* 3.3–5.8 p.p.m.). In contrast, inter­pretation of the ^13^C NMR spectrum was straightforward. In perfect agreement with the ^13^C NMR data of previously reported ammonium and azolium DPA salts (Huang *et al.*, 2011 ▶), the spectrum of the title compound displayed signals of the aromatic ring carbons at δ = 143.4, 139.5, 132.6, and 125.1 p.p.m. The two carbon resonances of the guanylurea cation were well separated at δ = 157.4 p.p.m. (C=O) and δ = 155.5 p.p.m. [NHC(NH_2_)_2_). IR bands in the range of 3200–3400 cm^−1^ were characteristic for the N—H valence vibrations in the guanylurea cation. A strong carbonyl band was observed at 1632 cm^−1^, whereas the band at 1532 cm^−1^ is characteristic for the nitro groups.

## Structural commentary   

**Figure 1:**
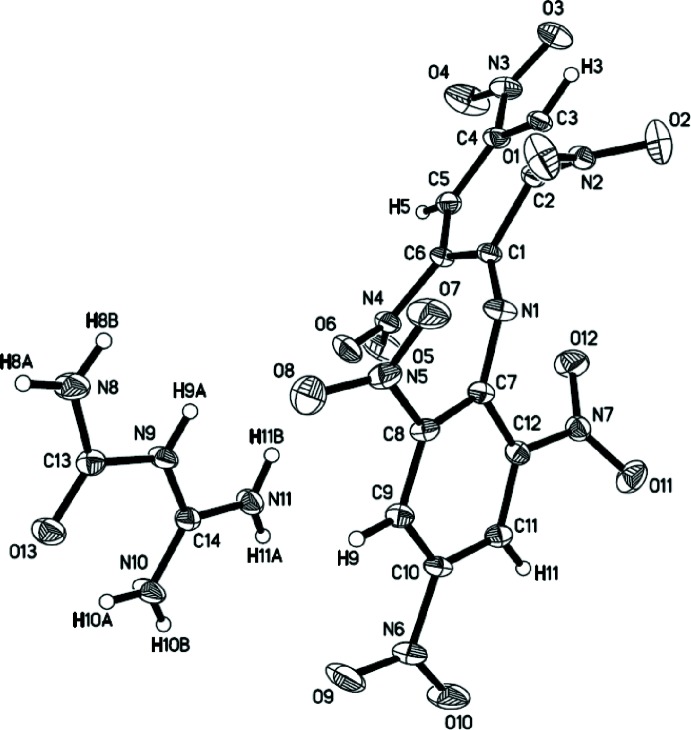
Mol­ecular structure of the title compound. Displacement ellipsoids represent 50% probability levels.

Single crystals obtained directly from the reaction mixture were found to be suitable for X-ray diffraction. The title compound crystallizes in the triclinic space group *P* 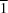. The crystal structure consists of mutually linked 1-carbamoylguanidinium cations and dipicryl­amide anions (Fig. 1 ▶). The angle C1—N1—C7 at the amide nitro­gen atom of the DPA^−^ anion is 131.66 (10)°, with C—N bond lengths of 1.3021 (15) (C1—N1) and 1.3403 (15) Å (C7—N1). These values are somewhat shorter than the C—N bond lengths in free dipicryl­amine (1.373, 1.375 Å; Huang *et al.*, 2011 ▶) but comparable to those reported for related azolium dipicryl­amides which have central C—N bond lengths in the range of 1.281–1.338 Å (Huang *et al.*, 2011 ▶). These values indicate delocalization of the nitro­gen lone pair on N1 in dipicryl­amide salts, thereby stabilizing the anion by strengthening the C1—N1 and C7—N1 bonds. As a structural consequence, not only is the central C1—N1—C7 angle widened, but there is also a significant elongation of the four C—C bonds adjacent to C1 and C7 (average 1.433 Å) as compared to the other aromatic C—C bonds (average 1.378 Å). The C—N bond lengths in the nearly planar (r.m.s. deviation = 0.0371 Å) 1-carbamoylguanidinium cation also indicate significant electron delocalization. The geometry around the carbon atom in the amidinium fragment NHC(NH_2_)_2_ is nearly trigonal-planar with N—C—N angles between 117.52 (11) and 121.48 (11)° and C—N distances in the very narrow range of 1.3064 (15)–1.3158 (15) Å. Overall, the structural parameters of the cation in the title compound do not differ significantly from those in 1-carbamoylguanid­in­ium sulfate, [H_2_NC(=O)NHC(NH_2_)_2_]_2_SO_4_ (Lotsch & Schnick, 2005 ▶).

## Supra­molecular features   

**Figure 2:**
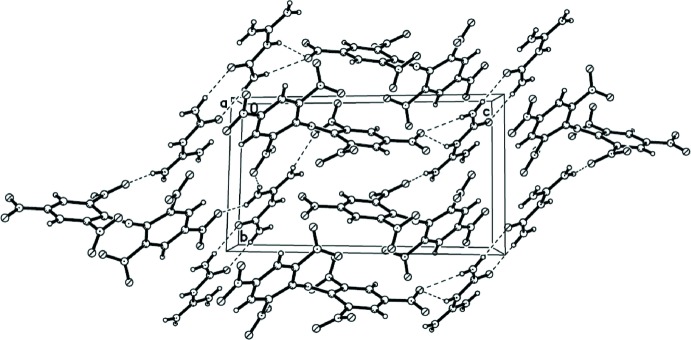
A packing diagram of the title compound. Dashed lines indicate N—H⋯O hydrogen-bonding inter­actions.

Both the cation and the anion comprise numerous sites capable of forming different types of hydrogen bonds. Thus it is not surprising that the crystal packing (Fig. 2 ▶) is controlled by an extensive hydrogen-bonding network (Table. 1 ▶). Six distinct N—H⋯O hydrogen bonds are found in the crystal packing of the title compound. First of all, pairs of cations are formed through dimerization *via* two N—H⋯O hydrogen bonds between the ureic fragments, which is also very typical for carb­oxy­lic amides. Furthermore, the NH_2_ groups in the amidinium fragments NHC(NH_2_)_2_ engage in four N—H⋯O hydrogen bonds to three different nitro groups of the DPA^−^ anion. The calculated density of 1.795 Mg m^−3^ is not only higher than the densities reported for other DPA-based salts (1.69–1.78 Mg m^−3^), but also much higher than the density of TNT (1.65 Mg m^−3^) (Huang *et al.*, 2011 ▶). The high density of the title compound can be traced back in large part to the hydrogen bonding in the crystal structure. The energetic properties (*e.g.* impact and friction sensitivity) of guanylurea dipicryl­amide have not been tested, but recent findings have shown that the impact sensitivities of various ammonium and azolium dipicryl­amides are in the range of that of the secondary explosive TNT (Huang *et al.*, 2011 ▶).

## Synthesis and crystallization   

***Cautionary note:** Dipicryl­amine and dipicryl­amide salts are potentially explosive and should be handled only in small amounts using proper safety equipment* (Klapötke, 2011 ▶).

Preparation of guanylurea dipicryl­amide: To a suspension of dipicryl­amine (1.0 g, 2.3 mmol) in 10 ml water were added two pellets of NaOH to give a dark red solution of sodium dipicryl­amide; 0.23 g (1.5 mmol) of guanylurea sulfate were added as solid, and the mixture was allowed to stand undisturbed at room temperature. After 10 d, 0.87 g (70%) dark-red crystals of the title compound had formed, which were isolated by filtration and dried in air. Analysis calculated for C_14_H_11_N_11_O_13_ (541.3 g/mol): C 31.06, H 2.05, N, 28.46; found: C 31.87, H 2.27, N 28.10%. IR (KBr pellet): *ν*_max_ 3403 (*vs*), 2171 (*w*), 1632 (*vs*), 1532 (*s*), 1402 (*vs*), 1270 (*s*), 1129 (*m*), 924 (*w*), 878 (*m*), 840 (*w*), 773 (*w*), 701 (*m*), 622 (*m*), 452 (*m*). ^1^H NMR (600 MHz, acetone-*d*_6_, 298 K): δ = 8.78 (*s*, 4 H, C_6_H_2_(NO_2_)_3_), *ca* 6.3–7.1 [vbr, 2 H, C(O)NH_2_], *ca* 3.3–5.8 [vbr, 5 H, NHC(NH_2_)_2_] p.p.m. ^13^C NMR (150.9 MHz, acetone-*d*_6_, 298 K): δ = 157.4 (C=O); 155.5 [NHC(NH_2_)_2_]; 143.4, 139.5, 132.6, 125.1 [C_6_H_2_(NO_2_)_3_] p.p.m.

## Refinement   

Crystal data, data collection and structure refinement details are summarized in Table 2 ▶. Positions and isotropic thermal parameters of hydrogen atoms were freely refined.

## Supplementary Material

Crystal structure: contains datablock(s) I, New_Global_Publ_Block. DOI: 10.1107/S1600536814017164/zl2596sup1.cif

Structure factors: contains datablock(s) I. DOI: 10.1107/S1600536814017164/zl2596Isup2.hkl

Supporting information file. DOI: 10.1107/S1600536814017164/zl2596Isup3.cml

CCDC reference: 1015895

Additional supporting information:  crystallographic information; 3D view; checkCIF report

## Figures and Tables

**Table 1:** Hydrogen-bond geometry (Å, °)

*D*—H⋯*A*	*D*—H	H⋯*A*	*D*⋯*A*	*D*—H⋯*A*
N8—H8*A*⋯O13^i^	0.87 (2)	2.11 (2)	2.9507 (17)	163.1 (18)
N8—H8*B*⋯O3^ii^	0.85 (2)	2.434 (19)	3.1495 (18)	142.4 (16)
N10—H10*A*⋯O13	0.86 (2)	1.98 (2)	2.6403 (16)	132.2 (17)
N10—H10*A*⋯O9^iii^	0.86 (2)	2.26 (2)	2.7991 (17)	120.7 (16)
N11—H11*A*⋯O1^iv^	0.859 (19)	2.222 (18)	2.7590 (16)	120.5 (15)
N11—H11*B*⋯O6	0.85 (2)	2.15 (2)	2.9833 (17)	167.7 (18)

Symmetry codes: (i) 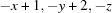; (ii) 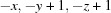; (iii) 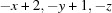; (iv) 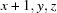.

**Table 2:** Experimental details

Crystal data	
Chemical formula	C_2_H_7_N_4_O^+^·C_12_H_4_N_7_O_12_^−^
*M*_r_	541.34
Crystal system, space group	Triclinic, *P* 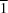
Temperature (K)	173
*a*, *b*, *c* (Å)	7.9764 (16), 8.6658 (17), 15.278 (3)
α, β, γ (°)	87.79 (3), 76.18 (3), 77.59 (3)
*V* (Å^3^)	1001.4 (3)
*Z*	2
Radiation type	Mo *K*α
μ (mm^−1^)	0.16
Crystal size (mm)	0.40 × 0.20 × 0.10

Data collection	
Diffractometer	Stoe IPDS 2T
Absorption correction	For a sphere [the interpolation procedure of Dwiggins (1975 ▶) was used with some modification]
*T*_min_, *T*_max_	0.861, 0.862
No. of measured, independent and observed [*I* > 2σ(*I*)] reflections	12316, 5347, 4600
*R*_int_	0.029
(sin θ/λ)_max_ (Å^−1^)	0.685

Refinement	
*R*[*F* ^2^ > 2σ(*F* ^2^)], *wR*(*F* ^2^), *S*	0.037, 0.097, 1.02
No. of reflections	5347
No. of parameters	387
H-atom treatment	All H-atom parameters refined
Δρ_max_, Δρ_min_ (e Å^−3^)	0.38, −0.24

Computer programs: *X-AREA* and *X-RED* (Stoe & Cie, 2002 ▶) and *SHELXS97*, *SHELXL97* and *XP* in *SHELXTL* (Sheldrick, 2008 ▶).

## supplementary crystallographic information

### Crystal data

**Table:**

C_2_H_7_N_4_O^+^·C_12_H_4_N_7_O_12_^−^	*Z* = 2
*M**_r_* = 541.34	*F*(000) = 552
Triclinic, *P*1	*D*_x_ = 1.795 Mg m^−^^3^
*a* = 7.9764 (16) Å	Mo *K*α radiation, λ = 0.71073 Å
*b* = 8.6658 (17) Å	Cell parameters from 15866 reflections
*c* = 15.278 (3) Å	θ = 2.4–29.6°
α = 87.79 (3)°	µ = 0.16 mm^−^^1^
β = 76.18 (3)°	*T* = 173 K
γ = 77.59 (3)°	Platelet, red
*V* = 1001.4 (3) Å^3^	0.40 × 0.20 × 0.10 × 0.40 (radius) mm

### Data collection

**Table:**

Stoe IPDS 2T diffractometer	5347 independent reflections
Radiation source: fine-focus sealed tube	4600 reflections with *I* > 2σ(*I*)
Graphite monochromator	*R*_int_ = 0.029
Detector resolution: 6.67 pixels mm^-1^	θ_max_ = 29.2°, θ_min_ = 2.4°
rotation method scans	*h* = −10→10
Absorption correction: for a sphere [the interpolation procedure of Dwiggins (1975) is used with some modification]	*k* = −11→10
*T*_min_ = 0.861, *T*_max_ = 0.862	*l* = −20→20
12316 measured reflections	

### Refinement

**Table:**

Refinement on *F*^2^	Primary atom site location: structure-invariant direct methods
Least-squares matrix: full	Secondary atom site location: difference Fourier map
*R*[*F*^2^ > 2σ(*F*^2^)] = 0.037	Hydrogen site location: inferred from neighbouring sites
*wR*(*F*^2^) = 0.097	All H-atom parameters refined
*S* = 1.02	*w* = 1/[σ^2^(*F*_o_^2^) + (0.0535*P*)^2^ + 0.3153*P*] where *P* = (*F*_o_^2^ + 2*F*_c_^2^)/3
5347 reflections	(Δ/σ)_max_ < 0.001
387 parameters	Δρ_max_ = 0.38 e Å^−^^3^
0 restraints	Δρ_min_ = −0.24 e Å^−^^3^

### Special details

**Table:**

Experimental. Absorption correction: interpolation using Int.Tab. Vol. C (1992) p. 523,Tab. 6.3.3.3 for values of muR in the range 0-2.5, and Int.Tab. Vol.II (1959) p.302; Table 5.3.6 B for muR in the range 2.6-10.0. The interpolation procedure of C.W.Dwiggins Jr (Acta Cryst.(1975) A31,146-148) is used with some modification.
Geometry. All e.s.d.'s (except the e.s.d. in the dihedral angle between two l.s. planes) are estimated using the full covariance matrix. The cell e.s.d.'s are taken into account individually in the estimation of e.s.d.'s in distances, angles and torsion angles; correlations between e.s.d.'s in cell parameters are only used when they are defined by crystal symmetry. An approximate (isotropic) treatment of cell e.s.d.'s is used for estimating e.s.d.'s involving l.s. planes.
Refinement. Refinement of *F*^2^ against ALL reflections. The weighted *R*-factor *wR* and goodness of fit *S* are based on *F*^2^, conventional *R*-factors *R* are based on *F*, with *F* set to zero for negative *F*^2^. The threshold expression of *F*^2^ > σ(*F*^2^) is used only for calculating *R*-factors(gt) *etc*. and is not relevant to the choice of reflections for refinement. *R*-factors based on *F*^2^ are statistically about twice as large as those based on *F*, and *R*- factors based on ALL data will be even larger.

### Fractional atomic coordinates and isotropic or equivalent isotropic displacement parameters (Å^2^)

**Table:**

	*x*	*y*	*z*	*U*_iso_*/*U*_eq_	
C1	0.13227 (14)	0.24457 (12)	0.39236 (7)	0.0151 (2)	
C2	−0.03965 (14)	0.21716 (13)	0.43593 (7)	0.0158 (2)	
C3	−0.11338 (14)	0.23130 (13)	0.52583 (8)	0.0171 (2)	
C4	−0.02176 (15)	0.28759 (13)	0.58079 (7)	0.0179 (2)	
C5	0.13785 (15)	0.33132 (13)	0.54440 (8)	0.0178 (2)	
C6	0.20813 (14)	0.31551 (13)	0.45315 (8)	0.0162 (2)	
C7	0.36230 (14)	0.18019 (13)	0.25658 (7)	0.0155 (2)	
C8	0.39833 (14)	0.26546 (13)	0.17498 (7)	0.0163 (2)	
C9	0.56417 (15)	0.25536 (13)	0.11987 (7)	0.0172 (2)	
C10	0.70261 (14)	0.14633 (13)	0.14019 (7)	0.0173 (2)	
C11	0.67718 (15)	0.04590 (13)	0.21192 (8)	0.0171 (2)	
C12	0.51165 (15)	0.06495 (13)	0.26863 (7)	0.0158 (2)	
C13	0.52565 (15)	0.81485 (13)	0.08734 (8)	0.0179 (2)	
C14	0.71331 (15)	0.61455 (13)	0.16006 (8)	0.0172 (2)	
N1	0.19803 (13)	0.19905 (13)	0.30867 (7)	0.0200 (2)	
N2	−0.13796 (12)	0.16126 (11)	0.37884 (6)	0.01692 (18)	
N3	−0.09042 (14)	0.29484 (14)	0.67591 (7)	0.0238 (2)	
N4	0.37044 (13)	0.37051 (12)	0.41873 (7)	0.0205 (2)	
N5	0.25570 (14)	0.37578 (12)	0.14648 (7)	0.0213 (2)	
N6	0.88031 (14)	0.14007 (13)	0.08619 (7)	0.0234 (2)	
N7	0.49658 (13)	−0.04504 (12)	0.34353 (7)	0.01838 (19)	
N8	0.35796 (15)	0.88985 (15)	0.09821 (8)	0.0259 (2)	
N9	0.55299 (13)	0.69662 (12)	0.15042 (7)	0.01906 (19)	
N10	0.85939 (14)	0.63735 (14)	0.10593 (8)	0.0261 (2)	
N11	0.71516 (15)	0.51081 (12)	0.22520 (7)	0.0215 (2)	
O1	−0.16781 (14)	0.24229 (13)	0.31524 (7)	0.0337 (2)	
O2	−0.18842 (15)	0.03875 (12)	0.39857 (7)	0.0314 (2)	
O3	−0.22256 (13)	0.23815 (13)	0.70773 (6)	0.0304 (2)	
O4	−0.01651 (15)	0.35404 (17)	0.72337 (7)	0.0422 (3)	
O5	0.47838 (14)	0.35606 (16)	0.46451 (8)	0.0397 (3)	
O6	0.38899 (13)	0.43379 (11)	0.34384 (6)	0.0274 (2)	
O7	0.10972 (12)	0.34437 (13)	0.16262 (7)	0.0317 (2)	
O8	0.29112 (15)	0.49270 (13)	0.10475 (8)	0.0367 (2)	
O9	0.90145 (13)	0.24434 (14)	0.03099 (7)	0.0352 (2)	
O10	0.99853 (12)	0.03311 (14)	0.09936 (8)	0.0354 (2)	
O11	0.58711 (14)	−0.17887 (11)	0.32959 (7)	0.0294 (2)	
O12	0.39664 (14)	0.00059 (12)	0.41533 (6)	0.0307 (2)	
O13	0.64763 (12)	0.84315 (11)	0.02764 (6)	0.02486 (19)	
H3	−0.223 (2)	0.207 (2)	0.5474 (11)	0.025 (4)*	
H5	0.199 (2)	0.369 (2)	0.5811 (11)	0.024 (4)*	
H8A	0.333 (3)	0.968 (2)	0.0628 (13)	0.037 (5)*	
H8B	0.277 (3)	0.865 (2)	0.1401 (13)	0.033 (5)*	
H9	0.583 (2)	0.321 (2)	0.0692 (11)	0.025 (4)*	
H9A	0.465 (3)	0.679 (2)	0.1898 (12)	0.031 (4)*	
H10A	0.852 (3)	0.705 (2)	0.0631 (13)	0.038 (5)*	
H10B	0.958 (3)	0.589 (3)	0.1157 (14)	0.046 (5)*	
H11	0.768 (2)	−0.030 (2)	0.2250 (11)	0.027 (4)*	
H11A	0.814 (3)	0.462 (2)	0.2352 (12)	0.031 (4)*	
H11B	0.621 (3)	0.502 (2)	0.2631 (13)	0.034 (5)*	

### Atomic displacement parameters (Å^2^)

**Table:**

	*U*^11^	*U*^22^	*U*^33^	*U*^12^	*U*^13^	*U*^23^
C1	0.0106 (5)	0.0169 (5)	0.0157 (5)	−0.0022 (4)	−0.0004 (4)	0.0022 (4)
C2	0.0119 (5)	0.0176 (5)	0.0176 (5)	−0.0037 (4)	−0.0027 (4)	0.0021 (4)
C3	0.0119 (5)	0.0191 (5)	0.0183 (5)	−0.0038 (4)	0.0000 (4)	0.0035 (4)
C4	0.0150 (5)	0.0215 (5)	0.0140 (5)	−0.0025 (4)	0.0013 (4)	0.0006 (4)
C5	0.0156 (5)	0.0189 (5)	0.0177 (5)	−0.0028 (4)	−0.0020 (4)	−0.0014 (4)
C6	0.0107 (4)	0.0180 (5)	0.0183 (5)	−0.0035 (4)	0.0004 (4)	0.0004 (4)
C7	0.0127 (5)	0.0192 (5)	0.0142 (5)	−0.0038 (4)	−0.0015 (4)	−0.0015 (4)
C8	0.0135 (5)	0.0187 (5)	0.0148 (5)	0.0004 (4)	−0.0027 (4)	−0.0009 (4)
C9	0.0165 (5)	0.0200 (5)	0.0138 (5)	−0.0037 (4)	−0.0012 (4)	0.0000 (4)
C10	0.0119 (5)	0.0221 (5)	0.0157 (5)	−0.0032 (4)	0.0009 (4)	−0.0018 (4)
C11	0.0132 (5)	0.0193 (5)	0.0179 (5)	−0.0013 (4)	−0.0036 (4)	−0.0011 (4)
C12	0.0156 (5)	0.0175 (5)	0.0144 (5)	−0.0043 (4)	−0.0032 (4)	0.0016 (4)
C13	0.0162 (5)	0.0205 (5)	0.0161 (5)	−0.0013 (4)	−0.0045 (4)	0.0004 (4)
C14	0.0141 (5)	0.0177 (5)	0.0186 (5)	−0.0025 (4)	−0.0024 (4)	0.0017 (4)
N1	0.0121 (4)	0.0303 (5)	0.0162 (4)	−0.0043 (4)	−0.0006 (3)	−0.0014 (4)
N2	0.0126 (4)	0.0196 (4)	0.0179 (4)	−0.0037 (3)	−0.0025 (3)	0.0027 (3)
N3	0.0185 (5)	0.0332 (5)	0.0160 (5)	−0.0033 (4)	0.0013 (4)	−0.0005 (4)
N4	0.0147 (4)	0.0227 (5)	0.0229 (5)	−0.0072 (4)	0.0018 (4)	−0.0041 (4)
N5	0.0184 (5)	0.0226 (5)	0.0190 (5)	0.0031 (4)	−0.0038 (4)	0.0003 (4)
N6	0.0138 (5)	0.0314 (5)	0.0214 (5)	−0.0033 (4)	0.0016 (4)	−0.0001 (4)
N7	0.0176 (5)	0.0184 (4)	0.0191 (4)	−0.0044 (3)	−0.0039 (4)	0.0028 (3)
N8	0.0152 (5)	0.0320 (6)	0.0261 (5)	0.0018 (4)	−0.0037 (4)	0.0071 (4)
N9	0.0110 (4)	0.0246 (5)	0.0190 (5)	−0.0024 (4)	−0.0006 (4)	0.0052 (4)
N10	0.0120 (5)	0.0321 (6)	0.0286 (5)	−0.0002 (4)	−0.0002 (4)	0.0146 (4)
N11	0.0164 (5)	0.0220 (5)	0.0238 (5)	−0.0031 (4)	−0.0025 (4)	0.0086 (4)
O1	0.0378 (6)	0.0385 (5)	0.0367 (5)	−0.0192 (4)	−0.0248 (5)	0.0205 (4)
O2	0.0435 (6)	0.0256 (5)	0.0346 (5)	−0.0194 (4)	−0.0177 (5)	0.0086 (4)
O3	0.0249 (5)	0.0429 (6)	0.0193 (4)	−0.0115 (4)	0.0059 (4)	0.0034 (4)
O4	0.0331 (6)	0.0761 (9)	0.0199 (5)	−0.0200 (6)	−0.0015 (4)	−0.0126 (5)
O5	0.0232 (5)	0.0655 (8)	0.0370 (6)	−0.0218 (5)	−0.0092 (4)	0.0016 (5)
O6	0.0252 (5)	0.0296 (5)	0.0252 (4)	−0.0133 (4)	0.0044 (4)	0.0024 (4)
O7	0.0154 (4)	0.0409 (5)	0.0363 (5)	0.0005 (4)	−0.0075 (4)	0.0043 (4)
O8	0.0336 (5)	0.0282 (5)	0.0433 (6)	0.0004 (4)	−0.0078 (5)	0.0145 (4)
O9	0.0218 (5)	0.0443 (6)	0.0327 (5)	−0.0087 (4)	0.0060 (4)	0.0125 (4)
O10	0.0134 (4)	0.0450 (6)	0.0387 (6)	0.0043 (4)	0.0011 (4)	0.0058 (5)
O11	0.0363 (5)	0.0191 (4)	0.0281 (5)	0.0028 (4)	−0.0070 (4)	0.0027 (3)
O12	0.0329 (5)	0.0293 (5)	0.0202 (4)	0.0006 (4)	0.0050 (4)	0.0062 (4)
O13	0.0184 (4)	0.0303 (5)	0.0208 (4)	−0.0003 (3)	−0.0007 (3)	0.0089 (3)

### Geometric parameters (Å, º)

**Table:**

C1—N1	1.3021 (15)	C13—N8	1.3295 (16)
C1—C2	1.4394 (15)	C13—N9	1.3980 (15)
C1—C6	1.4419 (16)	C14—N10	1.3064 (16)
C2—C3	1.3577 (16)	C14—N11	1.3158 (15)
C2—N2	1.4605 (15)	C14—N9	1.3615 (15)
C3—C4	1.3981 (17)	N2—O1	1.2167 (13)
C3—H3	0.924 (18)	N2—O2	1.2169 (14)
C4—C5	1.3880 (16)	N3—O4	1.2245 (16)
C4—N3	1.4241 (15)	N3—O3	1.2441 (15)
C5—C6	1.3728 (16)	N4—O5	1.2170 (15)
C5—H5	0.933 (17)	N4—O6	1.2398 (14)
C6—N4	1.4493 (15)	N5—O7	1.2184 (15)
C7—N1	1.3403 (15)	N5—O8	1.2248 (15)
C7—C12	1.4247 (16)	N6—O10	1.2205 (15)
C7—C8	1.4256 (16)	N6—O9	1.2228 (15)
C8—C9	1.3748 (16)	N7—O12	1.2146 (14)
C8—N5	1.4612 (15)	N7—O11	1.2237 (14)
C9—C10	1.3789 (16)	N8—H8A	0.87 (2)
C9—H9	0.943 (17)	N8—H8B	0.85 (2)
C10—C11	1.3770 (16)	N9—H9A	0.845 (19)
C10—N6	1.4506 (15)	N10—H10A	0.86 (2)
C11—C12	1.3755 (16)	N10—H10B	0.85 (2)
C11—H11	0.926 (17)	N11—H11A	0.859 (19)
C12—N7	1.4602 (15)	N11—H11B	0.85 (2)
C13—O13	1.2248 (15)		
			
N1—C1—C2	118.13 (10)	O13—C13—N9	121.68 (11)
N1—C1—C6	130.03 (10)	N8—C13—N9	113.95 (11)
C2—C1—C6	111.79 (10)	N10—C14—N11	121.48 (11)
C3—C2—C1	125.42 (10)	N10—C14—N9	121.00 (11)
C3—C2—N2	117.65 (10)	N11—C14—N9	117.52 (11)
C1—C2—N2	116.84 (10)	C1—N1—C7	131.66 (10)
C2—C3—C4	118.04 (10)	O1—N2—O2	123.85 (10)
C2—C3—H3	118.7 (10)	O1—N2—C2	117.73 (10)
C4—C3—H3	123.2 (10)	O2—N2—C2	118.40 (10)
C5—C4—C3	121.07 (10)	O4—N3—O3	122.46 (11)
C5—C4—N3	119.53 (11)	O4—N3—C4	119.22 (11)
C3—C4—N3	119.37 (10)	O3—N3—C4	118.32 (11)
C6—C5—C4	119.18 (11)	O5—N4—O6	123.58 (11)
C6—C5—H5	119.8 (10)	O5—N4—C6	119.33 (11)
C4—C5—H5	121.0 (10)	O6—N4—C6	117.07 (10)
C5—C6—C1	123.71 (10)	O7—N5—O8	123.41 (11)
C5—C6—N4	116.52 (10)	O7—N5—C8	118.60 (10)
C1—C6—N4	119.74 (10)	O8—N5—C8	117.94 (11)
N1—C7—C12	125.74 (10)	O10—N6—O9	124.42 (11)
N1—C7—C8	121.07 (10)	O10—N6—C10	118.28 (11)
C12—C7—C8	112.79 (10)	O9—N6—C10	117.29 (11)
C9—C8—C7	124.08 (10)	O12—N7—O11	123.96 (11)
C9—C8—N5	115.57 (10)	O12—N7—C12	118.82 (10)
C7—C8—N5	120.32 (10)	O11—N7—C12	117.22 (10)
C8—C9—C10	118.29 (11)	C13—N8—H8A	118.3 (13)
C8—C9—H9	121.0 (10)	C13—N8—H8B	121.2 (13)
C10—C9—H9	120.7 (10)	H8A—N8—H8B	120.4 (18)
C11—C10—C9	121.68 (11)	C14—N9—C13	125.47 (10)
C11—C10—N6	119.08 (10)	C14—N9—H9A	115.3 (12)
C9—C10—N6	119.23 (11)	C13—N9—H9A	118.9 (12)
C12—C11—C10	118.50 (10)	C14—N10—H10A	118.5 (13)
C12—C11—H11	119.0 (11)	C14—N10—H10B	119.0 (14)
C10—C11—H11	122.4 (11)	H10A—N10—H10B	122.4 (19)
C11—C12—C7	124.02 (10)	C14—N11—H11A	119.4 (12)
C11—C12—N7	115.00 (10)	C14—N11—H11B	121.4 (13)
C7—C12—N7	120.98 (10)	H11A—N11—H11B	118.3 (18)
O13—C13—N8	124.37 (11)		
			
N1—C1—C2—C3	167.76 (11)	C2—C1—N1—C7	−165.33 (12)
C6—C1—C2—C3	−9.85 (15)	C6—C1—N1—C7	11.8 (2)
N1—C1—C2—N2	−8.72 (15)	C12—C7—N1—C1	66.71 (19)
C6—C1—C2—N2	173.68 (9)	C8—C7—N1—C1	−121.14 (14)
C1—C2—C3—C4	4.74 (17)	C3—C2—N2—O1	125.71 (12)
N2—C2—C3—C4	−178.81 (10)	C1—C2—N2—O1	−57.53 (14)
C2—C3—C4—C5	1.49 (17)	C3—C2—N2—O2	−52.73 (15)
C2—C3—C4—N3	−176.28 (10)	C1—C2—N2—O2	124.03 (12)
C3—C4—C5—C6	−1.44 (17)	C5—C4—N3—O4	7.09 (18)
N3—C4—C5—C6	176.32 (10)	C3—C4—N3—O4	−175.11 (12)
C4—C5—C6—C1	−4.75 (17)	C5—C4—N3—O3	−171.84 (11)
C4—C5—C6—N4	177.02 (10)	C3—C4—N3—O3	5.96 (17)
N1—C1—C6—C5	−167.52 (12)	C5—C6—N4—O5	34.71 (16)
C2—C1—C6—C5	9.72 (15)	C1—C6—N4—O5	−143.59 (12)
N1—C1—C6—N4	10.66 (18)	C5—C6—N4—O6	−143.68 (11)
C2—C1—C6—N4	−172.10 (9)	C1—C6—N4—O6	38.02 (15)
N1—C7—C8—C9	177.97 (11)	C9—C8—N5—O7	147.05 (12)
C12—C7—C8—C9	−8.94 (16)	C7—C8—N5—O7	−34.82 (16)
N1—C7—C8—N5	0.00 (16)	C9—C8—N5—O8	−30.50 (16)
C12—C7—C8—N5	173.10 (10)	C7—C8—N5—O8	147.63 (12)
C7—C8—C9—C10	5.82 (17)	C11—C10—N6—O10	8.75 (17)
N5—C8—C9—C10	−176.13 (10)	C9—C10—N6—O10	−172.92 (12)
C8—C9—C10—C11	1.94 (17)	C11—C10—N6—O9	−170.40 (12)
C8—C9—C10—N6	−176.35 (10)	C9—C10—N6—O9	7.93 (17)
C9—C10—C11—C12	−5.54 (17)	C11—C12—N7—O12	145.75 (11)
N6—C10—C11—C12	172.75 (10)	C7—C12—N7—O12	−35.22 (16)
C10—C11—C12—C7	1.73 (17)	C11—C12—N7—O11	−34.09 (15)
C10—C11—C12—N7	−179.28 (10)	C7—C12—N7—O11	144.94 (11)
N1—C7—C12—C11	177.77 (11)	N10—C14—N9—C13	−2.99 (19)
C8—C7—C12—C11	5.06 (16)	N11—C14—N9—C13	177.65 (11)
N1—C7—C12—N7	−1.17 (17)	O13—C13—N9—C14	8.07 (19)
C8—C7—C12—N7	−173.87 (10)	N8—C13—N9—C14	−172.57 (12)

### Hydrogen-bond geometry (Å, º)

**Table:**

*D*—H···*A*	*D*—H	H···*A*	*D*···*A*	*D*—H···*A*
N8—H8*A*···O13^i^	0.87 (2)	2.11 (2)	2.9507 (17)	163.1 (18)
N8—H8*B*···O3^ii^	0.85 (2)	2.434 (19)	3.1495 (18)	142.4 (16)
N10—H10*A*···O13	0.86 (2)	1.98 (2)	2.6403 (16)	132.2 (17)
N10—H10*A*···O9^iii^	0.86 (2)	2.26 (2)	2.7991 (17)	120.7 (16)
N11—H11*A*···O1^iv^	0.859 (19)	2.222 (18)	2.7590 (16)	120.5 (15)
N11—H11*B*···O6	0.85 (2)	2.15 (2)	2.9833 (17)	167.7 (18)

Symmetry codes: (i) −*x*+1, −*y*+2, −*z*; (ii) −*x*, −*y*+1, −*z*+1; (iii) −*x*+2, −*y*+1, −*z*; (iv) *x*+1, *y*, *z*.
